# Triphenyltin Influenced Carotenoid-Based Coloration in Coral Reef Fish, *Amphiprion ocellaris*, by Disrupting Carotenoid Metabolism

**DOI:** 10.3390/toxics12010013

**Published:** 2023-12-22

**Authors:** Yan Zhang, Xingwei Cai, Yu Hou, Wenming Chen, Jiliang Zhang

**Affiliations:** 1 Ministry of Education Key Laboratory for Ecology of Tropical Islands, Key Laboratory of Tropical Animal and Plant Ecology of Hainan Province, College of Life Sciences, Hainan Normal University, Haikou 571158, China; 202311071300008@hainnu.edu.cn (Y.Z.); 20201071300047@hainnu.edu.cn (Y.H.); wenmingchen@aliyun.com (W.C.); 2 Hainan Academy of Ocean and Fisheries Sciences, Haikou 570206, China; caixw618@163.com; 3 Hainan Provincial Key Laboratory of Ecological Civilization and Integrated Land-Sea Development, Haikou 571158, China

**Keywords:** triphenyltin, *Amphiprion ocellaris*, carotenoids, oxidative damage

## Abstract

Triphenyltin (TPT), a kind of persistent pollutant, is prevalent in the aquatic environment and could pose a threat to coral reef fish. However, little is known about the toxicity of TPT on coral reef fish, especially regarding the representative characteristics of body coloration. Therefore, this study chose the clownfish (*Amphiprion ocellaris*) in order to investigate the effects of TPT exposure on its carotenoid-based body coloration under the environmentally relevant concentrations (0, 1, 10 and 100 ng/L). After TPT exposure for 60 d, the carotenoid contents were decreased and histological damage in the liver was found, shown as nuclear pyknosis and shift, lipid deposition and fibrotic tissue hyperplasia. Liver transcriptomic analysis showed that TPT exposure interfered with oxidative phosphorylation and fatty acid metabolism pathways, which related to carotenoids uptake and metabolism. Furthermore, TPT exposure led to oxidative damage in the liver, which is responsible for the changes in the antioxidant capacity of enzymes, including GSH, MDA, POD, CAT and T-SOD. TPT exposure also affected the genes (*Scarb1*, *CD36*, *Stard3* and *Stard5*) related to carotenoid absorption and transport, as well as the genes (*GstP1* and *Bco2*) related to carotenoid deposition and decomposition. Taken together, our results demonstrate that TPT influenced carotenoid-based coloration in coral reef fish by disrupting carotenoid metabolism, which complements the ecotoxicological effects and toxic mechanisms of TPT and provides data for the body color biology of coral reef fishes.

## 1. Introduction

Triphenyltin (TPT), a kind of organotin compound, is widely used as a plastic heat stabilizer, biocide, wood preservative and antifouling coating for ships [1,2]. Due to its excessive use, it may enter the aquatic environment through surface runoff resulting in the ecological pollution of aquatic areas [3,4]. In addition, TPT has been utilized as a biocide in marine antifouling coatings, which is one of the main reasons for its high concentration in the aquatic environment [5]. A large number of studies showed that TPT was detected in lots of aquatic environments [6]. For example, the concentrations of TPT ranged from 2.2 to 160 ng Sn/L in Xiamen coastal waters [7] and reached 37.2 ng Sn/L in the Jialing River of China [8]. The ecological risk assessment for TPT demonstrated that 57.1% of coastal waters in China were at risk [9]. Moreover, TPT was detected in many kinds of aquatic life, and the highest concentration seen reached 1079.9 ng/g (wet weight) [10].

Recently, multiple studies have reported that TPT exposure induced negative effects in fish. For example, TPT exposure for 21 days caused a loss of weight in marine medaka (*Oryzias melastigma*) [11]. It was found that there was behavioral hyperactivity after TPT exposure in marine medaka and behavioral hyperactivity may be caused by the interference of TPT with the neuroendocrine system [12]. TPT exposure also induced developmental toxicity in zebrafish (*Danio rerio*) embryos [13]. In addition, TPT exposure caused reproductive toxicity in fish. For example, TPT exposure for 5 weeks markedly suppressed the spawning frequency, spawned egg number, egg quality and gonad development in medaka (*Oryzias latipes*) [14]. In addition, organotin pollution is also widely distributed along the reef coastlines [15]. Thus, a potential target for TPT may be the coral reef ecosystem. Therefore, it is necessary to evaluate the effects of TPT on coral reef fish. According to our previous study, TPT exposure induced adverse effects on body coloration in adult male guppies [16]. Thus, we speculate that TPT exposure may also have adverse effects on body coloration in the clownfish.

Coral reef ecosystems are an important part of the marine ecosystem and are regarded as the “tropical rainforests of the ocean”. They have high ecological value and play a decisive role in maintaining the fishery economy, biodiversity, biological productivity and ecological balance [17]. Coral reefs, covering just 0.1% of the ocean surface, hold the highest species diversity of any marine ecosystem [18]. Coral reefs are subject to tremendous pressure due to environmental pollution [19]. Coral reef fishes are typical fishes in coral reef ecosystems and are crucial for maintaining the stability and sustainability of the coral reef ecosystem [20]. Meanwhile, they are important indicators for evaluating the condition of the coral reef ecosystem, and the decline in their quantity and diversity is often seen as an early warning of pressure on a coral reef ecosystem [21,22]. Furthermore, the clownfish are suggested to be used as a model species to study the impacts of marine pollution [23].

The body coloration of an animal has many functions, such as camouflage, deterrence, species recognition and courtship, which are crucial for survival and reproduction in fish [24,25,26,27]. Many studies have revealed that several pollutants can cause abnormal body coloration of fish. For example, orange/red and dark coloration in guppies (*Poecilia reticulata*) is affected by TPT [16]. The exposure of zebrafish embryos to ionizing radiation led to the significant loss of pigmentation [28]. After cyhalothrin-based pesticide exposure, a deeper pigmentation was observed in common carp (*Cyprinus carpio*) [29]. These results indicate that the body coloration of fish is sensitive to pollutants. Thus, the body coloration of fish might act as a crucial indicator for the ecotoxicological effects of pollutants.

The clownfish, a typical coral reef fish, has a bright body coloration. They are easy to identify and inhabit coral reefs. Therefore, in the present study, the clownfish were used to investigate the toxic effects of TPT exposure on carotenoid-based body coloration. It aims to reveal the physiological and molecular mechanism of TPT interfering with fish body coloration, enrich the understanding of ecotoxicological effects and toxic mechanisms of TPT, and provide data for the body color biology of coral reef fishes.

## 2. Materials and Methods

### 2.1. Chemicals

TPT chloride with a purity higher than 98% was purchased from Sigma Aldrich (St. Louis, MO, USA). It was gradually diluted with 95% ethanol to obtain 1, 10, and 100 ng/mL of stock solution TPT. The TPT stock solution was stored in sealed brown glass bottles at 4 °C. The stock solution was prepared every 7 days.

### 2.2. Fish Experimental Treatment

Fish used in this study were bought from an aquaculture farm situated in Wanning, Hainan Province, China. The fish with similar physical characteristics (body length: 2.63–3.17 cm, weight: 0.36–0.52 g) were chosen for exposure. A total of 25 fish were put in 15 L glass beakers randomly. The water quality was detected and maintained at a temperature of 29.1–31.1 °C, salinity of 32.85–34.85‰, a pH of 8.2–8.3, a dissolved oxygen level of above 6 mg/L and a light:dark cycle of 14:10 h. Feed was added to the beaker two times a day at 8 am and 6 pm, respectively. The amount of feed gradually increased as the fish grew. After 30 min of feeding, the stool at the bottom of the beaker were cleaned and 1/2 of the seawater was replaced. The stock solutions were added to the beakers to obtain TPT exposure concentrations of 1, 10, and 100 ng/L. And 1 μL/L 95% ethanol was added to the control beakers to a concentration equal to that of the exposure groups. Each exposure group or control group experiment was set up in triplicate. Total seawater was refreshed daily for each beaker at 7 am, and water with the same TPT concentrations was supplied. After 60 days of TPT exposure, the fish in each beaker were sampled.

### 2.3. Samples

The fish were anesthetized in ice water until they did not respond to contact. Then, the fish were rinsed with distilled water three times. The liver and intestinal tissues were carefully separated. Three liver samples were randomly selected and stored in 4% paraformaldehyde solution for liver histological detection, six liver samples were frozen for liver antioxidant enzyme activity analysis, and three liver samples were frozen for liver transcriptome analysis. Six intestinal and liver samples were used to analyze the expression levels of genes related to carotenoid metabolism.

### 2.4. Carotenoid Measurement

Carotenoids were extracted according to the method described by Andrade et al. (2019) [30]. Astaxanthin, lutein, β-carotene and canthaxanthin were measured, respectively. The standard stock solution with a mass concentration of 1 mg/mL was prepared with acetonitrile solution. The standard stock solution was diluted with acetonitrile to 1.0, 2.0, 5.0, 10.0, 25.0, 50.0 and 100.0 μg/mL to obtain the standard working solution, and it was used immediately after preparation. An Agilent Zorbax Bonus RP column (2.1 × 50 mm, 1.8 µm) was used for liquid chromatography. The specific experimental conditions are shown in Table S1. Methods are as follows, preparing standard working solution for chromatographic analysis, drawing standard curves with the concentrations of 4 compounds as the horizontal axis and the peak area response values as the vertical axis, and performing linear regression to obtain the standard curve linear regression equation. Samples were filtered into a brown chromatographic vial through 0.22 μm organic filter membrane for chromatographic analysis. The peak area of each compound was measured, and content was determined based on area and standard curve equation.

### 2.5. Histological Detection

Liver tissues were stripped carefully from fish. After being fixed in 4% paraformaldehyde solution for 24 h, the liver samples were dehydrated with alcohol, washed with xylene, embedded in paraffin and sliced to 5 μm. Then, slices were stained with hematoxylin and eosin (H&E) according to the method described by Plonka et al. (2009) [31]. Six liver regions were randomly selected for microscope observation (bar = 20 μm or 50 μm).

### 2.6. Antioxidant Capacity Analysis

The protocol for antioxidant capacity analysis was according to the instructions of Nanjing Jiancheng Kit for total antioxidant capacity (T-AOC), total superoxide dismutase (T-SOD), peroxidase (POD), catalase (CAT), glutathione (GSH) and malondialdehyde (MDA).

### 2.7. Bioinformatics Analysis

Total RNA extraction and transcriptome analysis were performed by Nuo He Bio-Tech. The ds-cDNA (double-strand complementary DNA) was fragmented using the Covaris system, followed by end repair and poly(A) tail addition to the short fragments. Sequencing connectors were then ligated, and AM Pure XP beads were used for the purification and selection of fragments of approximately 200 bp. The cDNA library was subsequently obtained through PCR (polymerase chain reaction) amplification.

The Agilent 2100 system was used to determine the insert size of the library, while qPCR (quantitative real-time PCR) was used to accurately quantify the effective concentration of the library (requiring a concentration > 2 nmol/L). Sequencing was performed on the Illumina Hiseq TM2000 platform, resulting in the acquisition of raw reads. These raw reads were aligned with the NR (Non-Redundant Protein Sequence Database), NT (Nucleotide Sequence Database), Swiss Prot, Prarm, GO (Gene Ontology), KOG (clusters of orthologous groups for eukaryotic complete genomes) and KEGG (Kyoto Encyclopedia of Genes and Genomes) databases using the BLAST+ 2.14.0 tool.

The comparison of the clean reads was conducted using the RSEM v1.3.3 software, and the expression levels were quantified using FPKM (fragments per kb per million fragments). The DESeq2 method was applied to identify differentially expressed genes (DEGs) based on the read count data obtained from the gene expression analysis. A filtering threshold of *p* < 0.05 and a |log2 Fold Change| > 1 value were used to determine significance.

To further analyze the DEGs, they were mapped to the corresponding terms in the GO database. The number of genes in each GO term was calculated, and the function and corresponding genes associated with the differentially expressed genes were determined. Hypergeometric distribution testing was performed with a significance level of *p* < 0.05.

Additionally, the significant enrichment analysis of the DEGs was conducted using the KEGG database. Hypergeometric tests with the significance level of *p* < 0.05 were performed, aiming to identify the pathways in which the DEGs were significantly enriched compared to annotated genes. The unit of analysis was the KEGG pathway.

To perform GSEA (gene set enrichment analysis), we began by preparing the gene expression data and associated sample labels. Next, relevant gene sets were selected from databases like GO, KEGG and KOG. The genes were ranked in the dataset according to their differential expressions. Then, the enrichment score was computed, which measures the cumulative deviation of gene ranks within each gene set. The statistical significance of these scores were assessed by comparing them to randomly permuted gene sets to construct a null distribution. *p*-values and false discovery rates (FDR) were calculated to determine the significance of enrichment. The results were interpreted by analyzing the enriched gene sets, associated pathways or processes. And the results were visualized by using enrichment plots or heatmaps to depict the enrichment patterns across different sample groups.

### 2.8. Quantitative Real-Time PCR Analysis

According to the protocol of Hou et al. (2022) [16], the RNA was extracted and cDNA were synthesized. The primers used in the present study are shown in Table S2. Brilliant III Ultra-Fast SYBR Green QPCR Master Mix (Agilent Technologies, Santa Clara, CA, USA) and AriaMX real-time PCR system (Agilent Technologies, Santa Clara, CA, USA) were utilized for qRT–PCR. The analyzed genes related to carotenoid included Scavenger receptor class B type I (*Scarb1*), Cluster determinant 36 (*CD36*), StAR-related lipid transfer (START) domain containing 3 (*Stard3*), StAR-related lipid transfer (START) domain containing 5 (*Stard5*), Apolipoprotein D (*ApoD*), Glutathione S-transferase P1 (*Gstp1*), Beta-carotene15,15′-monooxygenase (*Bcom1*) and Beta-carotene 9′,10′-oxygenase (*Bco2*).

### 2.9. Statistical Analysis

The data were presented as the means ± standard error (SE). According to our previous research, gene expression was analyzed with the Pair-Wise Fixed Reallocation Randomization Test^©^ [32]. The other data were analyzed by one-way analysis of variance (ANOVA) followed by Tukey’s post hoc test (SPSS Version 13.0, SPSS Inc., New York, NY, America).

## 3. Results

### 3.1. Carotenoid Contents

**Figure 1:**
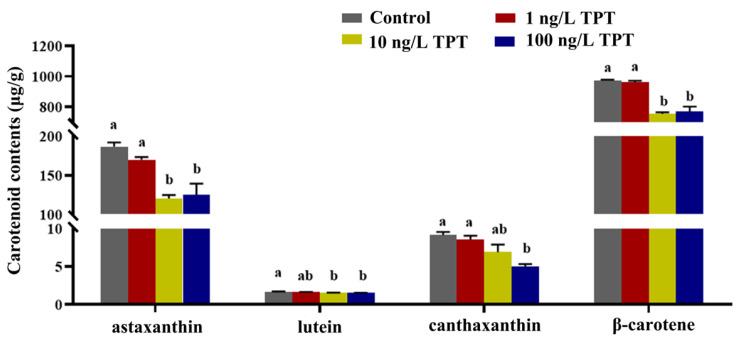
Carotenoids (*n* = 6) in the clownfish after TPT (0, 1, 10 or 100 ng/L) exposure for 60 d. Data are shown as the mean ± SE; the means of groups not sharing a common letter are significantly different at *p* < 0.05.

Carotenoid contents, including astaxanthin, lutein, β-carotene and canthaxanthin, decreased after TPT exposure (Figure 1). Compared with the control group, the contents of astaxanthin, lutein and β-carotene were significantly decreased only in the 10 and 100 ng/L TPT groups. The contents of canthaxanthin were significantly decreased only in the 100 ng/L TPT group (Figure 1).

### 3.2. Liver Histology

**Figure 2:**
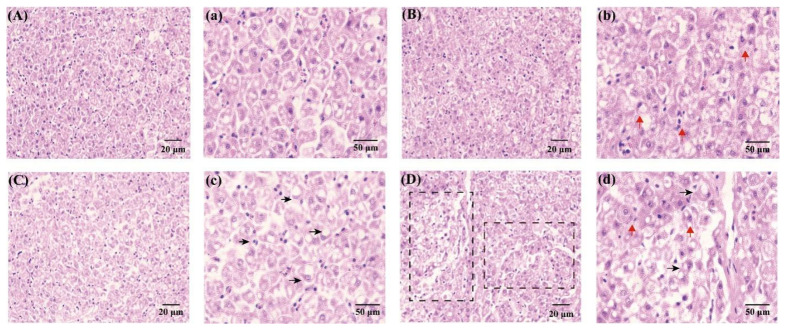
Hepatocytes in the clownfish after TPT (0, 1, 10 or 100 ng/L) exposure for 60 d. (**A**–**d**) Hepatocytes in the control (**A**,**a**), 1 (**B**,**b**), 10 (**C**,**c**) and 100 ng/L TPT (**D**,**d**) groups (20 μm and 50 μm, respectively); red arrows indicate nuclear pyknosis and shift; black arrows indicate lipid deposition; fibrotic tissue hyperplasia is circled in black squares.

After 60 days of TPT exposure, damage to hepatic cells was observed (Figure 2A). The H&E staining of hepatic tissue results showed that the hepatocytes were regular and complete in the control group (Figure 2A,a). With the increase in TPT concentrations, the extent of hepatocyte damage became enlarged. In the 1 and 10 ng/L TPT groups, hepatocyte enlargement, karyopyknosis and nuclear translocation were seen (Figure 2B–c), while in the 100 ng/L TPT group, the disappearance of hepatic cell nuclei and fibrotic tissue hyperplasia were observed (Figure 2D,d).

### 3.3. Transcriptome Analysis

**Figure 3:**
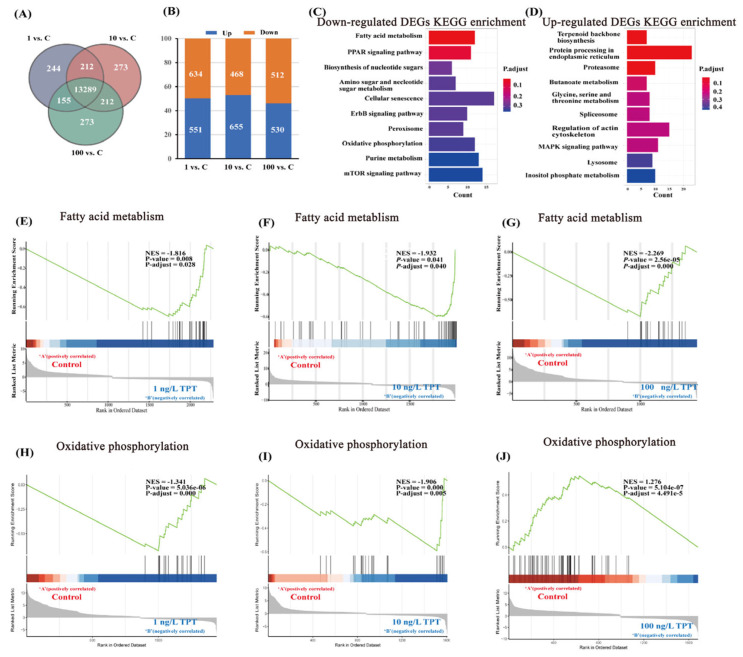
Hepatic transcriptome in the clownfish after TPT (0, 1, 10 or 100 ng/L) exposure for 60 d. (**A**) Venn diagram of differentially expressed genes between each concentration of TPT exposure group and control group; (**B**) shows the number of significantly different genes between each concentration of TPT exposure group and control group; (**C**,**D**) show the KEGG enrichment results of significantly up-regulated genes and significantly down-regulated genes in the TPT exposure group and the control group. (**E**–**G**) show the GSEA analysis results of fatty acid metabolic pathways in each TPT exposure group; (**H**–**J**) show the GSEA analysis results of oxidative phosphorylation pathways in each TPT exposure group.

A Venn diagram depicts the DEGs between each concentration in the TPT exposure group (TPT) and the control group (Con) (Figure 3A). In the TPT vs. Con groups, a total of 13,259 DEGs were identified (Figure 3A). Additionally, the number of significantly different genes were analyzed between the TPT exposure group and the control group (Figure 3B).

The results of KEGG enrichment revealed that significantly down-regulated genes were primarily associated with fatty acid metabolism and oxidative phosphorylation pathways (Figure 3C). Conversely, significantly up-regulated genes were primarily linked to terpenoid backbone biosynthesis and protein processing in endoplasmic reticulum (Figure 3D).

GSEA analysis results for the fatty acid metabolism and oxidative phosphorylation pathways were conducted in the 1, 10 and 100 ng/L TPT exposure groups, respectively (Figure 3E–J). TPT exposure inhibited the fatty acid metabolism pathway (Figure 3E–G). In the 1 and 10 ng/L TPT exposure groups, the oxidative phosphorylation pathway was inhibited, while in the 100 ng/L TPT exposure group, the oxidative phosphorylation pathway was up-regulated (Figure 3H–J).

### 3.4. Hepatic Oxidative Damage

**Figure 4:**
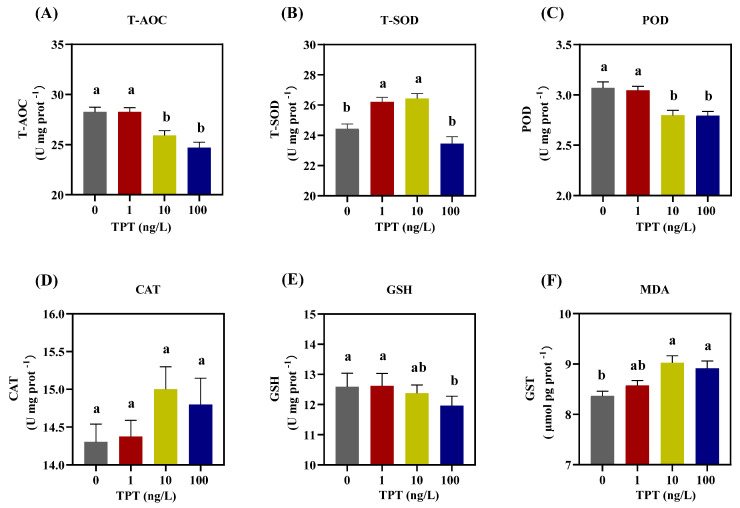
Hepatic oxidative enzymes in the clownfish after TPT (0, 1, 10 or 100 ng/L) exposure for 60 d. (**A**–**F**) Samples (*n* = 6) of T-AOC (**A**), T-SOD (**B**), POD (**C**), CAT (**D**), GSH (**E**) and MDA (**F**). Data are shown as the mean ± SE; the means of groups not sharing a common letter are significantly different at *p* < 0.05.

TPT exposure affected the antioxidant capacity of enzymes in the liver of the clownfish, including T-AOC, T-SOD, POD, CAT, GSH and MDA. Compared with the control group, there was no significant change in the levels of T-AOC and POD in the 1 ng/L TPT group, while a significant decrease was found in the 10 and 100 ng/L TPT groups (Figure 4A,C). TPT exposure increased the levels of T-SOD in the 1 and 10 ng/L TPT groups, while no significant change was found in the 100 ng/L TPT group (Figure 4B). No significant change in the levels of CAT was found in the 1, 10 and 100 ng/L TPT groups (Figure 4D). And no significant change in the levels of GSH was found in the 1 and 10 ng/L TPT groups, while a significant reduction was found in the 100 ng/L TPT group (Figure 4E). Meanwhile, compared with the control group, TPT exposure increased the levels of MDA in the 10 and 100 ng/L TPT groups, which reached significance (*p* < 0.05), while no significant change was found in the 1 ng/L TPT group (Figure 4F).

### 3.5. Genes Related to Carotenoid Absorption and Transport

**Figure 5:**
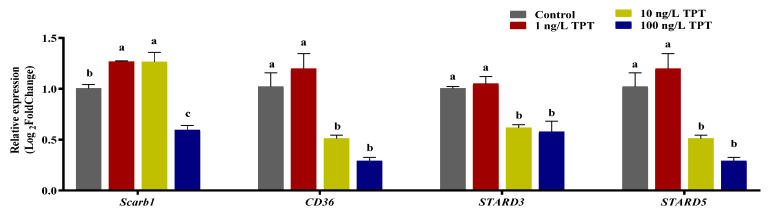
Expression of genes related to the carotenoid (*n* = 6) absorption and transport in the clownfish after TPT (0, 1, 10 or 100 ng/L) exposure for 60 d. The *Scarb1* and *CD36* related to carotenoid absorption were analyzed in the intestine, and the *Stard3* and *Stard5* related to carotenoid transport were analyzed in the liver. Data are shown as the mean ± SE; the means of groups not sharing a common letter are significantly different at *p* < 0.05.

TPT exposure affected the expression level of genes (*Scarb1*, *CD36*, *Stard3* and *Stard5*) related to carotenoid absorption and transport in the intestine or the liver. Compared with the control group, TPT exposure significantly increased the expression of *Scarb1* in the intestine in the 1 and 10 ng/L TPT groups, while it was significantly reduced in the 100 ng/L TPT group (Figure 5). Compared to the control group, no significant change in the expression of *CD36* in the intestine was found in the 1 ng/L TPT group, while significantly decreased in the 10 and 100 ng/L TPT groups (Figure 5). No significant change in the expression of *Stard3* and *Stard5* in the liver were found in the 1 ng/L TPT group, while the expression of the *Stard3* and *Stard5* significantly decreased in the 10 and 100 ng/L TPT groups compared with the control (Figure 5).

### 3.6. Genes Related to Carotenoid Deposition and Decomposition

**Figure 6:**
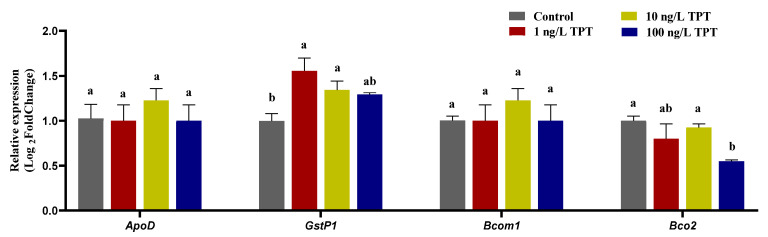
Expression of genes related to the carotenoid (*n* = 6) deposition and decomposition in the clownfish after TPT (0, 1, 10 or 100 ng/L) exposure for 60 d. The *GstP1* and *ApoD* genes related to carotenoid deposition were analyzed in the liver, and the *Bcom1* and *Bco2* genes related to carotenoid decomposition were analyzed in liver. Data are shown as the mean ± SE; the means of groups not sharing a common letter are significantly different at *p* < 0.05.

TPT exposure also affected the expression level of genes (*GstP1* and *Bco2*) related to carotenoid deposition and decomposition in the liver. Compared with the control group, TPT exposure increased the expression of *GstP1*, which reached significance in the 1 and 10 ng/L TPT groups, while no significant change was found in 100 ng/L TPT group (Figure 6). TPT exposure reduced the expression of *Bco2* only in the 100 ng/L TPT group (Figure 6). No significant change in the expression of *ApoD* and *Bcom1* was found in the 1, 10 and 100 ng/L TPT groups compared with the control group (Figure 6).

## 4. Discussion

A variety of pollutants have been reported to cause abnormal body coloration in fishes. For example, the sexually attractive orange-yellow coloration was reduced in the adult male guppy after antiandrogenic pesticide exposure [33]. The deposition of carotenoids in the viviparous amarillo fish (*Girardinichthys multiradiatus*) is influenced by embryonic exposure to low concentrations of an organophosphorus insecticide [34]. Cyhalothrin-based pesticide exposure induced a higher incidence of deeper pigmentation in common carp [29]. In our study, the carotenoid coloration was reduced after TPT exposure in the adult clownfish, which provided new evidence for abnormal body coloration induced by pollutants.

### 4.1. Carotenoid Contents

Carotenoids are antioxidants, which are added to feed to alleviate stress damage in animals [35,36]. Meanwhile, the carotenoid trade-off hypothesis describes the trade-off between carotenoids in animal skin color and antioxidant stress [37,38,39]. In animals, the carotenoids are prioritized for oxidative stress damage, while excessive carotenoids are deposited in the skin, hair or scales for decoration [40]. We speculate that the decrease in astaxanthin, lutein, β-carotene and canthaxanthin observed might be associated with oxidative damage. Meanwhile, the results of liver damage and oxidative damage after TPT exposure in this study have proved that the reduction in carotenoid content was related to oxidative damage, which can be explained by the carotenoid trade-off hypothesis [41].

### 4.2. Liver Histology

Studies have shown that TPT accumulates mainly in the liver of fish [42]. Thus, liver is the main sites for carotenoid metabolism. Thus, liver tissues were observed in order to investigate whether they are affected by TPT. The present study demonstrated that the extent of hepatocyte damage was enlarged with the increasing of TPT concentrations. The results were consistent with those of a previous study [2]. In addition, the liver is involved in the uptake, synthesis, packaging and secretion of lipids [42]. TPT exposure caused the changes in liver tissues, and we speculate that this result may be caused by abnormal fatty acid metabolism and oxidative phosphorylation. Liver transcriptome analysis showed that fatty acid metabolism and oxidative phosphorylation were inhibited, which supported our hypothesis.

### 4.3. Transcriptome Analysis

GSEA analysis results indicated that TPT exposure affected the fatty acid metabolic pathway in the livers of clownfish. The liver is able to utilize fatty acids as an internal energy source through oxidation [43], meeting the organismic energy needs. It seems that in the 1 ng/L TPT exposure group, the up-regulation of fatty acid metabolism serves as a strategy to compensate for less intake resulting from TPT exposure. Conversely, in the 10 and 100 ng/L TPT exposure groups, medium–high TPT exposure induced liver function impairment and inhibited the fatty acid metabolic pathway. The results were consistent with the results of carotenoid contents and liver histology, which showed that the carotenoid contents were decreased, liver tissues were damaged and the fatty acid metabolic pathway was inhibited. The liver is also responsible for the synthesis of some lipoproteins, apolipoproteins and enzymes for lipoprotein metabolism [43]. Carotenoids are colourful liposoluble pigments [44]. Therefore, the result of the inhibition of the fatty acid metabolic pathway was associated with the decrease in carotenoid contents.

Oxidative phosphorylation is a vital biochemical process that takes place in the inner mitochondrial membrane of eukaryotic or in the cytoplasm of prokaryotes cells, which releases large amounts of energy. A study has shown that atrazine exposure interfered with the appetite of common carp [44]. In the present study, the oxidative phosphorylation pathway was inhibited after 1 and 10 ng/L TPT exposure. An explanation for this may be that the inhibition of the oxidative phosphorylation pathway is related to the suppression of appetite by TPT. Interestingly, the oxidative phosphorylation pathway was induced in the 100 ng/L TPT exposure group, which may be a compensatory response due to inadequate intake.

Thus, TPT exposure interfered with oxidative phosphorylation and fatty acid metabolism pathways related to carotenoids uptake and metabolism, leading to the decrease in carotenoid contents.

### 4.4. Hepatic Oxidative Damage

In order to explore the oxidative damage of liver, we detected the activities of antioxidant-related enzymes. The results showed that the activities of T-AOC, POD and T-SOD were decreased after TPT exposure. T-AOC is the sum of antioxidant substances and the ability of organisms to resist peroxide damage [45,46]. POD exists in almost all species and plays a crucial role in detoxifying in organisms [47]. T-SOD, which exists in animals, plants and microorganisms, is an important antioxidant and plays a crucial role in maintaining a balance between oxidation and antioxidants [48,49]. Moreover, the biological actions of carotenoids include antioxidant activity [50]. Therefore, their reduction may be associated with a decrease in carotenoid contents. Carotenoids serve as antioxidants involved in remediating oxidative damage in the liver, resulting in the drop of carotenoid contents.

Moreover, the GSH is the most powerful intracellular antioxidant, which plays a role in the detoxification of a variety of electrophilic compounds and peroxides via catalysis by glutathione-S-transferases (GST) and glutathione peroxidases (GPx) [51]. The GSH values decreased slowly after the TPT exposure. These results may be due to the TPT exposure at low concentrations not affecting the GSH contents, while at high concentrations it inhibited the GSH values in the clownfish. In addition, GSH is an important metabolic regulator. Thus, we speculated that the synthesis of GSH is also related to the suppression of appetite by TPT.

In addition, MDA levels are commonly known as the marker of oxidative stress and the antioxidant status [52]. MDA also affects the permeability and fluidity of plasma membrane and is damaging to the structure and function of cells [53]. The MDA level was increased following TPT exposure. According to the histological results in this study, the reason for this result may be that the liver is further affected by oxidative stress. CAT is the first line of defense against reactive oxygen species (ROS), which catalyze the decomposition of H_2_O_2_ and protect cells from ROS and H_2_O_2_ toxicity [54]. In the present study, the activities of CAT in the livers of the clownfish were increased, which may be a response to the oxidative damage induced by the TPT exposure.

### 4.5. Genes Related to Carotenoid Metabolism

The genes *Scarb1*, *CD36*, *Stard3*, *Stard5*, *ApoD*, *GstP1*, *Bcom1* and *Bco2*, which play important roles in carotenoid metabolism, were found to be affected by TPT exposure. It has been reported that *Scarb1* and *CD36* are involved in the regulation of carotenoid pigments in body color [55]. They play a key role in carotenoid absorption [56]. In this study, TPT exposure significantly increased the expression of *Scarb1* in the intestine in the 1 and 10 ng/L TPT groups, while significantly reducing that in the 100 ng/L TPT group. The expression of *CD36* in the intestine was similar to that of *Scarb1*. It might be that TPT exposure at low concentrations appears to promote carotenoids absorption, while at high concentrations, it inhibits carotenoid absorption. *Stard3* and *Stard5* are responsible for cholesterol transport [57]. TPT exposure increased the expression of *Scarb1* and *Scarb5* in the liver in the 1 ng/L TPT group, while it was significantly reduced in the 10 and 100 ng/L TPT groups. It might be that TPT exposure at low concentrations appears to promote carotenoid transport, while at high concentrations, it inhibits carotenoid transport.

The downregulation of liver detoxifying enzyme Gstp1 is partly responsible for the exacerbation of liver injury in mice [58,59]. This study found that TPT exposure increased the expression of *Gstp1* in the liver and caused liver damage in clownfish. Thus, an explanation may be that the up-regulation of *Gstp1* reduces liver damage in clownfish. In mammals, *Bcom1* and *Bco2* are involved in the decomposition of carotenoids [60]. According to results in this study, the downregulation of *Bco2* in the liver may relate to carotenoids acting as antioxidants against liver oxidative damage. Rassart et al. reported that *Apod* is closely associated with antioxidation and antiapoptosis in organisms [61]. In this study, there were no significant differences in the expression levels of *ApoD* and *Bcom1* in the liver between the TPT-exposed group and the control group, which might indicate that other mechanisms are interfering with the expression of *ApoD* and *Bcom1.* Taken together, the disruption of these genes might be related to the decrease in carotenoid contents observed in this study.

## 5. Conclusions

**Figure 7:**
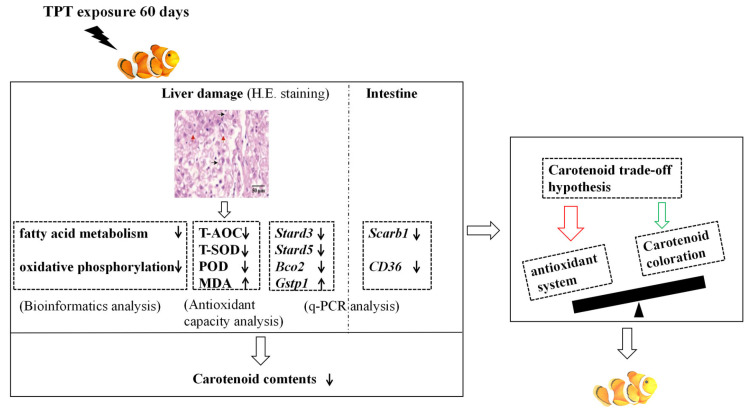
Schematic diagram of the molecular mechanism of TPT-induced carotenoid-based coloration of clownfish. The techniques used in this study are H&E staining, q-PCR, bioinformatics, etc. The thin black arrows represents fall or rise.

In conclusion, as shown in Figure 7, TPT exposure leads to histological damage in clownfish livers. And when oxidative phosphorylation and fatty acid metabolism pathways are inhibited, oxidative damage occurs in the liver and the expression levels of genes related to the carotenoid metabolism are induced in the intestine or the liver. Then, the results cause an imbalance between the antioxidant system and the carotenoid coloration, resulting in the carotenoid-based coloration in the clownfish being lighter. The present study highlights the harmful effects of TPT exposure on *A. ocellaris* and emphasizes the importance of reducing the use of organic persistent pollutants and finding alternatives to minimize harm to aquatic life.

## Data Availability

The original data presented in the study are included in the article and Supplementary Materials; further inquiries can be directed to the corresponding author.

## Supplementary Materials

The following supporting information can be downloaded at: https://www.mdpi.com/article/10.3390/toxics12010013/s1, Table S1: Liquid chromatographic conditions; Table S2: Primers for real-time PCR.
