# Supplementary material for: Triphenyltin Influenced Carotenoid-Based Coloration in Coral Reef Fish, Amphiprion ocellaris, by Disrupting Carotenoid Metabolism
Source: Toxics. 2023 Dec 22;12(1):13. doi: 10.3390/toxics12010013 (PMC10820653; doi:10.3390/toxics12010013)
Supplement: Supplementary file 1 [file toxics-12-00013-s001.zip › toxics-2755403-supplementary materials.pdf]

Table S1. Liquid chromatographic conditions.

Column temperature	Sample size	Flow rate	Detection wavelength	Elution time
28 °C	2 μL	1.0 mL/min	470 nm	20 min

Mobile phase A is water and mobile phase B is acetonitrile.
Gradient elution procedure:
0 ~ 5 min, 78 ~ 85 % mobile phase B;
5 ~ 6.5 min, 85 ~ 100 % mobile phase B;
6.5 ~ 10 min, 100 % B;
10 ~ 12 min, 100 ~ 78 % mobile phase B.

Table S2. Primers for real-time PCR.

Gene	Forward primer	Reverse primer	Gene ID
Scarb1	TGGCGAGACGACTCAACATT	TGGGTCGATCATTGTGTTCTTTG	XM_023279004.2
Bco1	GGGGAAACCAGCTACGAACA	CTGGGTTGCGGTTGACTACT	XM_023299893.2
Bcmo1	GCTTAACTCCGGCTGTGTCT	GACTGTATCTCTCCGTGCCG	NM_001328495.1
CD36	CGCATGTGGAAACACTGTCG	CAATTCACGTCCGTTGCCAG	XM_023265863.2
ApoD	GCCTCTTCACTGGCCTTCAT	GGGGTTGCTCTCACAATCCA	XM_035951069.1
GstP1	TCTGCAGGCGGAACTGAC	ATCACCCAGAGAGGCGGC	NM_131734.3
Stard3	AGAGACGAGTCTGGGTGGAA	CGATGATCCCCTCACCCTTG	XM_023294238.2
Stard5	ACAGAACGTTAGCTCGCTCC	TGACGCTACAGCGGTTCAAT	XM_023264138.2
gapdh	CGGAATCAACGGATTCGGTC	TCACTTCGCCCTTAAAGCG	XM_023263785.2
